# Evaluation of a panel of spermatological methods for assessing reprotoxic compounds in multilayer semen plastic bags

**DOI:** 10.1038/s41598-020-79415-7

**Published:** 2020-12-17

**Authors:** M. Schulze, F. Schröter, M. Jung, U. Jakop

**Affiliations:** 1 Institute for Reproduction of Farm Animals Schönow, Bernauer Allee 10, 16321 Bernau, Germany; 2 Department of Cardiovascular Surgery, Heart Center Brandenburg, Brandenburg Medical School “Theodor-Fontane”, Faculty of Health Brandenburg, Ladeburger Str. 17, 16321 Bernau, Germany

**Keywords:** Animal biotechnology, Toxicology, Reproductive biology

## Abstract

The increase of fertility performance in sows is one of the biggest achievements in pig production over the last 30 years. Nevertheless, pig farms using artificial insemination (AI) repeatedly experienced in recent year’s fertility problems with dramatic consequences due to toxic compounds from plastic semen bags. In particular, bisphenol A diglycidyl-ether (BADGE) present in multilayer plastic bags can leach into the semen and could affect the functionality of the spermatozoa. Former studies could not find any alterations in spermatozoa based on the exposure to BADGE. The aim of the study was to evaluate effects of BADGE on boar spermatozoa using an extended panel of spermatological methods. In spring 2019, a large drop in farrowing rates from 92.6 ± 2.3% to 63.7 ± 11.1% in four sow farms in Croatia was detected. In migration studies, BADGE could be identified as a causal toxic compound and leached into the extended semen in concentration of 0.37 ± 0.05 mg/L. Detailed spermatological studies showed that significant predictors for effects on spermatozoa were different levels of motility and kinematic data after a prolonged storage time, thermo-resistance test (prolonged incubation time), mitochondrial activity, membrane integrity and fluidity. No serious effects were observed for sperm morphology and DNA fragmentation. These results provide new insights into the development of a new quality assurance concept for a detailed spermatological examination during testing of plastic materials for boar semen preservation. It could be shown that boar spermatozoa are an excellent biosensor to detect potential toxicity and fertility-relevant compounds.

## Introduction

From April to May 2019 a strong drop in non-return rates in four sow farms in Croatia was detected. In most of the litters farrowed, a reduction of the litter size was reported. The affected sow farms were supplied from one AI center located in Croatia with semen packaged in multilayer MEDI NOVA plastic bags. All boar semen doses as well as all the materials used to process these doses passed the quality assurance (QA) program of the AI center without showing any abnormal results. There was no evidence of other factors capable of affecting the overall fertility of semen produced in the AI center such as water and feed quality, health status, semen extender quality and poor semen quality. Furthermore, no evidence of factors negatively affecting fertility on the female side were identified during the investigation of the problem. This included health status, feed quality and absence of mycotoxins, seasonality and poor insemination techniques. Once the toxic batches of semen bags were identified and eliminated, the farms returned to normal fertility rates.

During storage of boar semen at 16 to 18 °C in multilayer plastic bags for several days^1^, compounds (plasticizers and adhesives) present in the plastic bags can leach into the semen^2–4^. The manufacturing of products for boar semen collection^5^, processing and insemination^6^ requires a large variety of plastic materials. In order to provide hygienic and safe environments for boar semen, a careful selection of the raw materials and a suitable design of the products is essential. Particularly, the plastics utilized for the fabrication of semen-packaging units such as bottles, tubes, flat packs or blisters must be evaluated with special diligence to ensure high sperm quality^7^ even after direct exposure to the plastic materials during storage. The absence of any detrimental effects on the fertilizing potential of stored semen is of upmost importance for any semen-packaging unit. Therefore, exhaustive tests of the raw material proving its compliance with sperm physiology are mandatory before manufacturing and commercializing any product.

Nerin et al. (2014) could not find any sperm defects and alterations when her group performed routine sperm quality analyses and in vitro penetration tests in toxic plastic bags. Only in vivo fertility studies confirmed that adhesives were indeed the main cause for the reproductive failure in 41 sow farms in Spain^4^. Bisphenol A diglycidyl-ether (BADGE) and two diol derivatives were identified as toxic compounds and affected probably the early embryonic development and blastocyst implantation. BADGE is used frequently in combination with polyamines, polyaminoamides and acid anhydrides in the productions of plastics^8^.

The aim of the present study was, therefore, to evaluate the efficacy of an extended panel of spermatological methods to detect reprotoxic effects of BADGE and its influence on boar spermatozoa prior to its use in sow herds. Such a sensitive assay may have application for implementing a QA concept for animal breeding companies. An additional aim was to demonstrate how a detailed spermatological examination could be important for testing of any plastic materials being used for boar semen preservation.

## Results

### Data from affected pig farms in croatia

The results shown in Table 1 summarize the fertility data recorded 8 weeks prior (Before), 8 weeks after (After) and 8 weeks during (During) the usage of the MEDI NOVA bags. Farms were located in Croatia and housed between 1300 and 2000 PIC Camborough or L03 sows (Pig Improvement Company, Hendersonville, USA). The semen used for breeding was obtained from one AI center located in Croatia without any obvious deviations in standard sperm quality parameters (> 80% total motility and > 75% morphologically normal spermatozoa). Health status, water, semen extender and feed quality (absence of mycotoxins), seasonality, vaccines, pharmaceutical products, deworming procedures, insemination techniques and other possible stressors were carefully evaluated and did not change during the investigated timeframe. The comparison of the periods before and after the usage of the semen bags revealed statistical differences (*P* < 0.001) in farrowing rate, total born and born alive pigs per sow compared to the period during the usage. The percentage of abortions did not differ significantly (*P* > 0.05) between periods.

**Table 1:** Percentage of farrowing rate in sows and litter size of piglets in four sow farms in Croatia before, during and after using MEDI NOVA bags.

Period	Sows (n)	Farms (n)	Farrowing rate (%)	Total born alive (n)	Total born/sow (n)	Born alive/sow (n)	Abortions (%)
Before	2,412	4	92.6 ± 2.3^a^	32,746	16.3 ± 0.2^a^	14.6 ± 0.2^a^	0.2 ± 0.2
During	2,627	4	63.7 ± 11.1^b^	16,991	10.8 ± 1.9^b^	9.9 ± 1.7^b^	0.04 ± 0.1
After	2,737	4	89.7 ± 0.9^a^	35,934	16.0 ± 0.2^a^	14.6 ± 0.2^a^	0.2 ± 0.3
*P*-Value	n.a.	n.a.	< 0.0001	n.a.	< 0.0001	< 0.0001	0.44

Significant differences between the time-points are marked by the superscript index letters (a & b).

Values are represented as mean ± SD. *P* values: ANOVA/Fisher LSD Test.

### Migration study in semen bags

**Figure 1:**
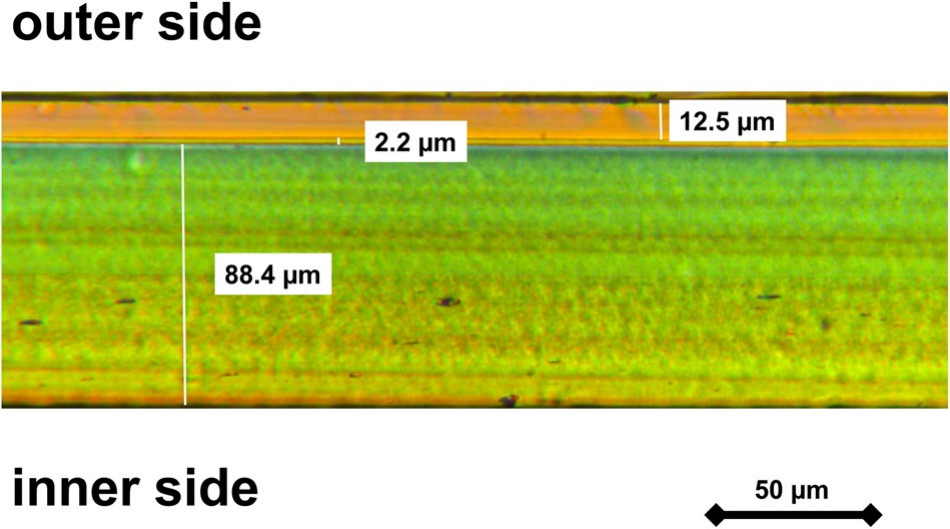
Microtome sections (Leitz polarizing microscope) of the multilayer MEDI NOVA bag. The adhesive layer is 2.2 µm wide. Underneath is the inner thinner layer (88.4 µm) that comes into contact with the extended semen. The outer layer is 12.5 µm wide (*Source* Fraunhofer Institute for Process Engineering and Packaging, Freising, Germany).

**Figure 2:**
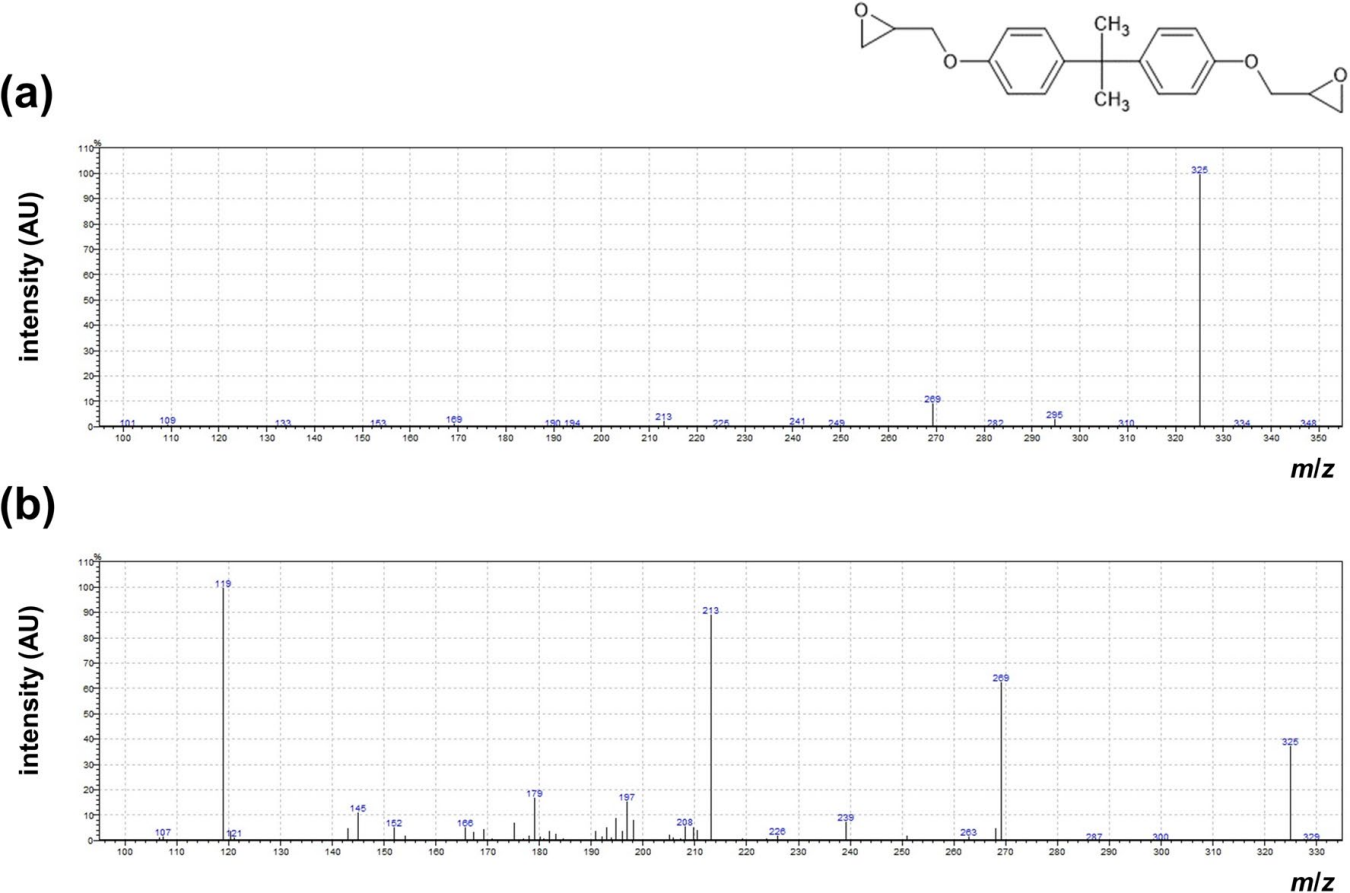
GC–MS/MS mass spectra of a migration solution from multilayer MEDI NOVA bags [molar mass: 340 g × mol^−1^ (**a**) and 325 g × mol^−1^ (**b**)].

A representative polarizing microscopy image of the MEDI NOVA bags and the different layers is shown in Fig. 1. The detected masses in the GC–MS/MS of the migration solution from multilayer MEDI NOVA bags could be assigned to the decomposition products of the substance BADGE (Fig. 2) reaching a concentration of 0.37 ± 0.05 mg/L (mean ± SD). Other selected adhesives and/or plasticizers (e.g. acetyltributylcitrate (ATBC), butyl benzyl phthalate (BBP), bis(2-ethylhexyl) adipate (DEHA), diethylhexyl phthalate (DEHP), diisobutyl phthalate (DIBP), diisodecyl phthalate (DIDP), diisononyl phthalate (DINP), dibutyl phthalate (DBP), 1,2-cyclohexane dicarboxylic acid diisononyl ester (DINCH), diethyl phthalate (DEP), diisoheptyl phthalate (DIHP), di-n-octyl phthalate (DNOP), dimethyl phthalate (DMP), diisobutyl adipate (DIBA), triisobutyl phosphate (TIBP), diethyl adipate (DEA) and dibutyl adipate (DBA)) were not detectable.

### Spermatological analysis

**Figure 3:**
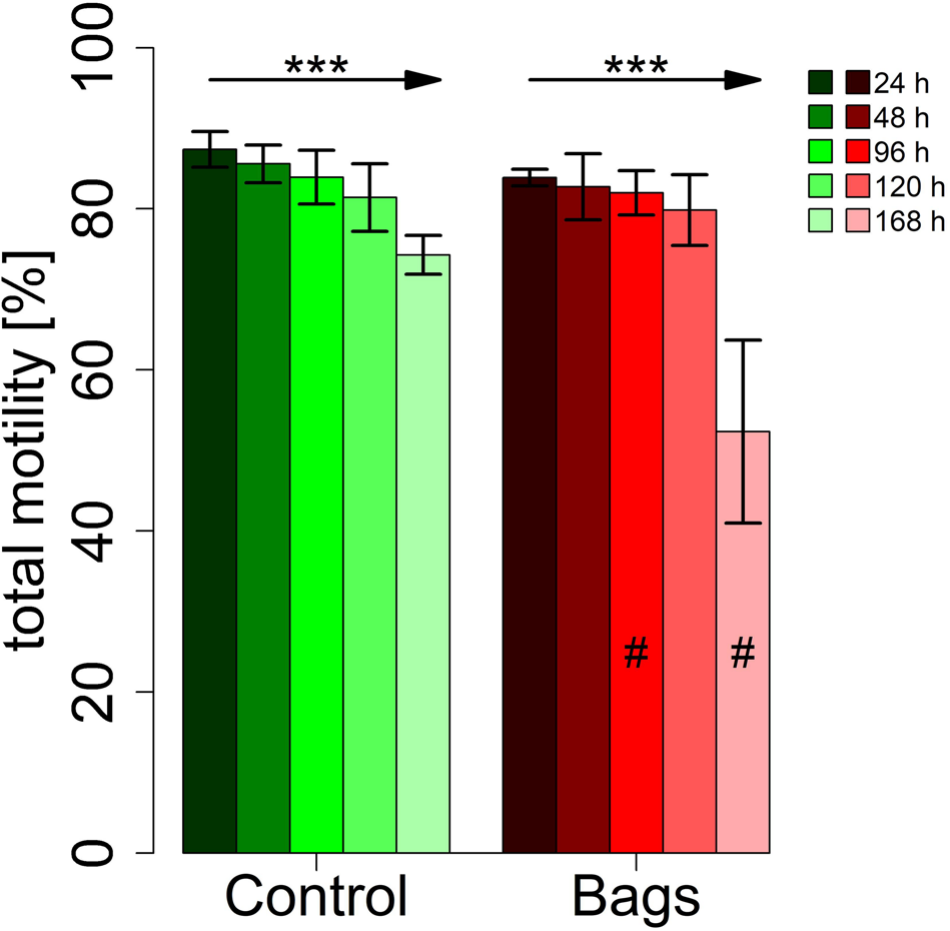
Percentage of motile spermatozoa (%) in sixteen pooled ejaculates (4 × 4 ejaculates) from 16 mature Pietrain boars after 24, 48, 96, 120 and 168 h of semen storage. Correlation between time and motility: ***P < 0.001, significance of differences between MEDI NOVA bags and control ^#^*P* < 0.05.

**Figure 4:**
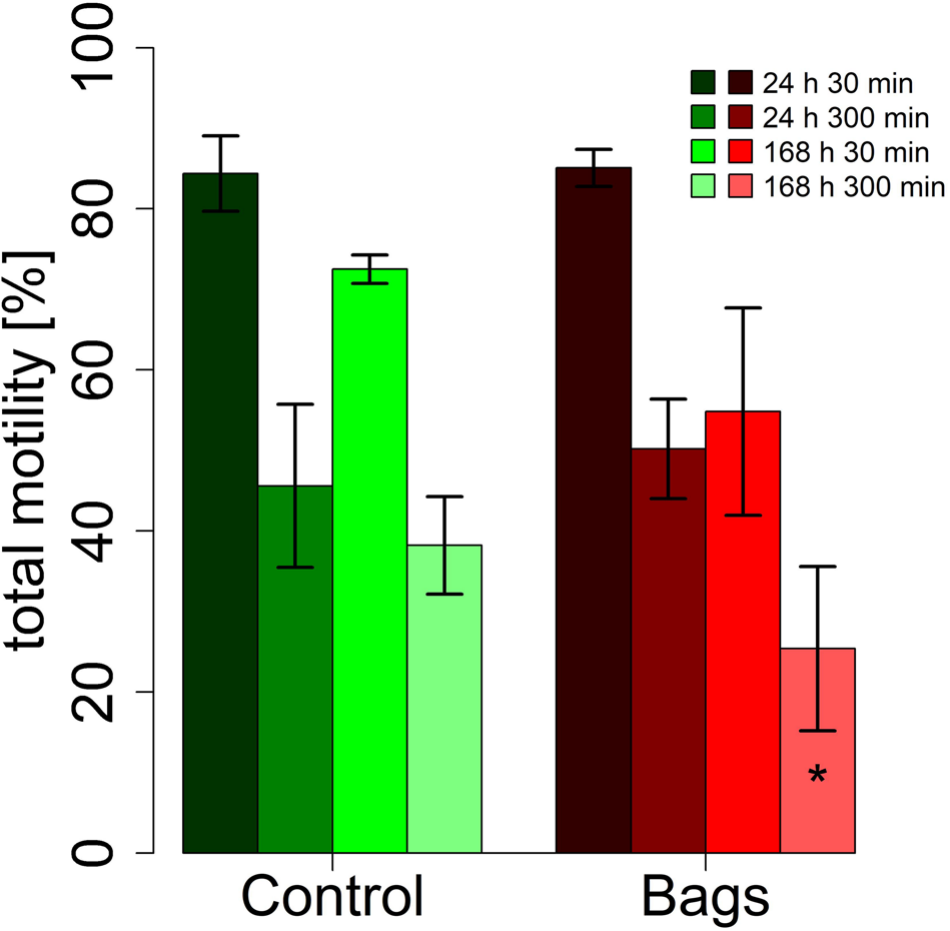
Thermo-resistance test of boar spermatozoa in sixteen pooled ejaculates (4 × 4 ejaculates) from 16 mature Pietrain boars after 24 and 168 h of semen storage. Significance of differences between MEDI NOVA bags and control *P < 0.05.

**Figure 5:**
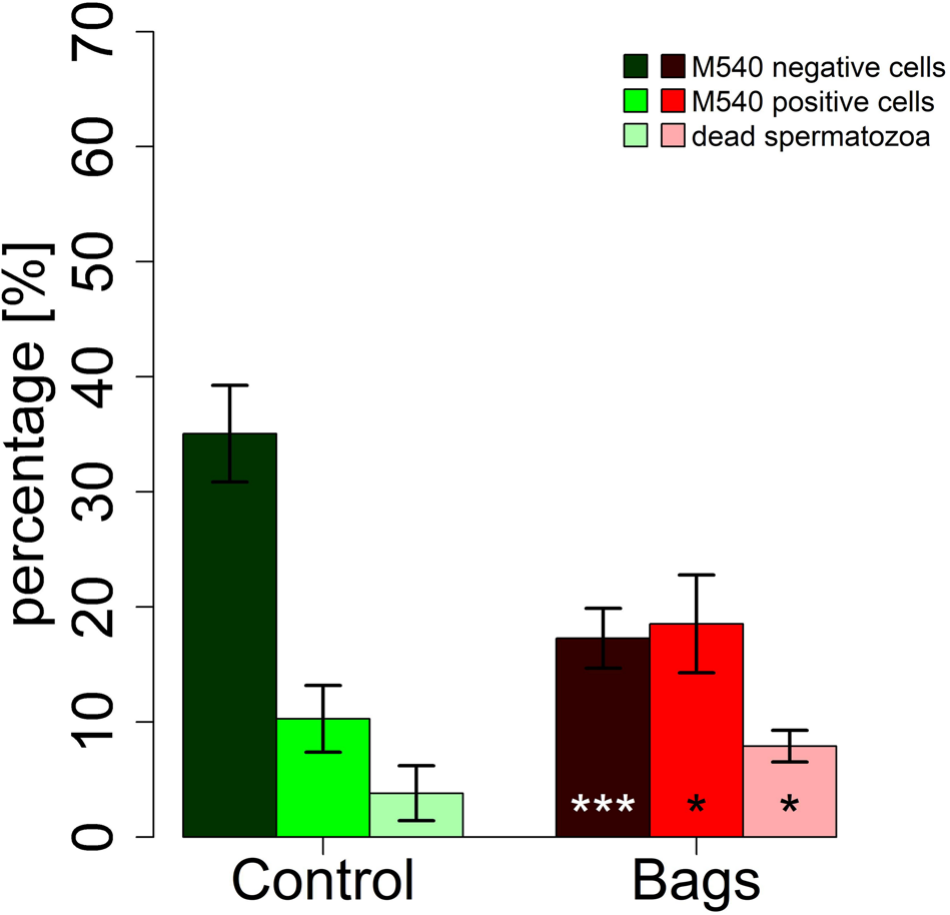
Membrane fluidity of boar spermatozoa in sixteen pooled ejaculates (4 × 4 ejaculates) from 16 mature Pietrain boars after 168 h of semen storage. Significance of differences between MEDI NOVA bags and control. ***P < 0.001, *P < 0.05.

**Figure 6:**
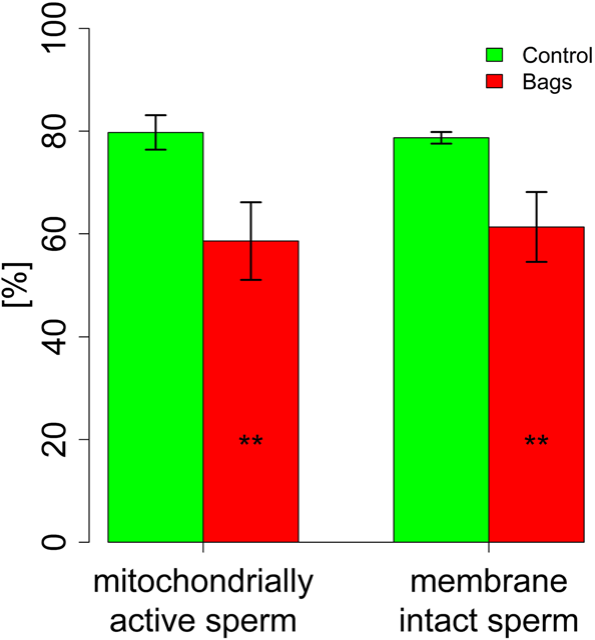
Mitochondrial activity and plasma membrane and acrosome integrity of boar spermatozoa in sixteen pooled ejaculates (4 × 4 ejaculates) from 16 mature Pietrain boars after 168 h of semen storage. Significance of differences between MEDI NOVA bags and control. *P < 0.05.

The total sperm motility decreased significantly over the course of 7 days in the bags as well as in the controls (*P* < 0.001). Total sperm motility was lower in the bags at 96 h (*P* = 0.014) and 168 h (*P* = 0.029) compared to the controls (Fig. 3). The differences between bags and control were further demonstrated by the thermo-resistance test, which showed a lower total motility in the bags (*P* = 0.037) after 168 h storage with subsequent 300 min incubation at 38 °C (Fig. 4). The total motility decreased in both groups with length of incubation at 38 °C or storage time (Figs. 3, 4). The bags contained fewer M540 negative cells (*P* = 0.001, low lipid disorder) and more M540 positive cells (*P* = 0.04, high lipid disorder) and more dead spermatozoa (*P* = 0.021, Fig. 5). The control group contained significantly more mitochondrially active (79.7 ± 3.4% *vs.* 58.6 ± 7.6%, *P* = 0.005, Fig. 6) and membrane and acrosome intact spermatozoa (78.7 ± 1.1% *vs.* 61.3 ± 6.8%, *P* = 0.011, Fig. 6) than the test group. An increase in sperm morphology abnormalities was observed after 24 h of semen storage (*P* = 0.001). There was no change in DFI between control and test group (Table 2). Several different kinematic parameters after 10 min incubation at 38 °C (Table 3) and after thermo-resistance test (Table 4) were negatively affected by storage in the bags compared to the controls especially after prolonged storage and incubation times.

**Table 2:** Morphologically intact spermatozoa and DNA defragmentation index of boar spermatozoa in sixteen pooled ejaculates (4 × 4 ejaculates) from 16 mature Pietrain boars in control and MEDI NOVA bags.

Characteristics	Storage time (h)	Control	Bags	*P*-value
Morphologically intact spermatozoa (%)	24	84.1 ± 3.4	81.0 ± 3.0	0.001
Morphologically intact spermatozoa (%)	72	84.3 ± 4.4	83.4 ± 1.7	0.683
DNA defragmentation index (%)	72	1.53 ± 0.36	1.55 ± 0.66	0.981

Values are represented as mean ± SD.

**Table 3:** Sperm kinematic data of CASA analysis after 10 min at 38 °C of boar spermatozoa in sixteen pooled ejaculates (4 × 4 ejaculates) from 16 mature Pietrain boars in control and MEDI NOVA bags.

Characteristics	Storage time (h)	Control	Bags	*P*-value
Progressive motility (%)	24	*84.8* ± *2.1*	*80.3* ± *0.4*	*0.021*
Distance curved line (µm)		69.3 ± 5.8	68.2 ± 0.7	0.692
Distance straight line (µm)		*22.7* ± *0.9*	*21.1* ± *1.3*	*0.016*
Distance average path (µm)		34.4 ± 2.1	33.3 ± 1.2	0.430
Velocity curved line (µm/s)		153.7 ± 11.5	150.4 ± 3.0	0.581
Velocity straight line (µm/s)		*54.7* ± *2.1*	*51.5* ± *2.0*	*0.021*
Velocity average path (µm/s)		78.4 ± 2.4	76.0 ± 1.6	0.247
Beat cross frequency (Hz)		25.8 ± 1.0	24.1 ± 0.7	0.056
Amplitude of lateral head displacement (µm)		1.36 ± 0.11	1.34 ± 0.05	0.689
Progressive motility (%)	48	*82.5* ± *3.1*	*79.7* ± *3.2*	*0.006*
Distance curved line (µm)		*69.4* ± *2.2*	*62.8* ± *2.4*	*0.018*
Distance straight line (µm)		22.0 ± 2.4	22.1 ± 1.6	0.917
Distance average path (µm)		33.8 ± 1.6	31.6 ± 1.4	0.062
Velocity curved line (µm/s)		*155.4* ± *5.3*	*140.4* ± *5.2*	*0.017*
Velocity straight line (µm/s)		54.9 ± 3.6	54.5 ± 1.9	0.738
Velocity average path (µm/s)		*78.8* ± *2.4*	*73.8* ± *1.9*	*0.035*
Beat cross frequency (Hz)		24.3 ± 1.3	23.1 ± 1.0	0.053
Amplitude of lateral head displacement (µm)		*1.38* ± *0.07*	*1.25* ± *0.06*	*0.07*
Progressive motility (%)	96	*81.0* ± *4.1*	*78.6* ± *3.6*	*0.004*
Distance curved line (µm)		64.7 ± 1.2	60.6 ± 2.6	0.093
Distance straight line (µm)		23.4 ± 2.5	22.4 ± 1.5	0.300
Distance average path (µm)		*32.6* ± *1.1*	*30.8* ± *1.7*	*0.070*
Velocity curved line (µm/s)		145.8 ± 5.4	138.6 ± 5.6	0.135
Velocity straight line (µm/s)		57.7 ± 3.2	56.8 ± 2.7	0.464
Velocity average path (µm/s)		*76.5* ± *2.4*	*73.9* ± *2.8*	*0.017*
Beat cross frequency (Hz)		*23.7* ± *1.5*	*22.0* ± *1.3*	*0.013*
Amplitude of lateral head displacement (µm)		1.28 ± 0.07	1.21 ± 0.05	0.164
Progressive motility (%)	120	77.5 ± 5.3	75.8 ± 3.9	0.383
Distance curved line (µm)		59.5 ± 1.6	54.4 ± 4.6	0.063
Distance straight line (µm)		22.4 ± 3.0	22.3 ± 1.9	0.886
Distance average path (µm)		30.5 ± 1.8	28.7 ± 2.3	0.139
Velocity curved line (µm/s)		*135.8* ± *5.2*	*124.2* ± *10.8*	*0.036*
Velocity straight line (µm/s)		56.1 ± 5.7	54.1 ± 3.8	0.127
Velocity average path (µm/s)		*72.6* ± *3.9*	*67.4* ± *4.4*	*0.016*
Beat cross frequency (Hz)		22.4 ± 1.6	20.4 ± 0.5	0.074
Amplitude of lateral head displacement (µm)		1.17 ± 0.05	1.12 ± 0.13	0.376
Progressive motility (%)	168	*70.0* ± *2.7*	*47.0* ± *10.1*	*0.018*
Distance curved line (µm)		*52.7* ± *2.7*	*30.7* ± *7.1*	*0.006*
Distance straight line (µm)		*20.4* ± *2.2*	*12.4* ± *1.8*	*0.025*
Distance average path (µm)		*26.1* ± *1.3*	*15.3* ± *2.8*	*0.010*
Velocity curved line (µm/s)		*123.8* ± *9.3*	*73.5* ± *16.8*	*0.002*
Velocity straight line (µm/s)		*53.1* ± *4.9*	*33.4* ± *3.8*	*0.011*
Velocity average path (µm/s)		*65.1* ± *4.7*	*39.6* ± *6.2*	*0.004*
Beat cross frequency (Hz)		*18.4* ± *0.8*	*11.1* ± *2.7*	*0.022*
Amplitude of lateral head displacement (µm)		*1.11* ± *0.09*	*0.74* ± *0.17*	*0.009*

Significant differences are marked with italics.

**Table 4:** Sperm kinematic data of CASA analysis after thermo-resistance test (30 and 300 min at 38 °C) of boar spermatozoa in sixteen pooled ejaculates (4 × 4 ejaculates) from 16 mature Pietrain boars in control and MEDI NOVA bags.

Characteristics	Storage time (h)	Incubation time					
		30 min			300 min		
		Control	Bags	*P*-value	Control	Bags	*P*-value
Progressive motility (%)	24	81.3 ± 6.1	82.5 ± 2.7	0.717	39.2 ± 9.9	44.5 ± 5.8	0.202
Distance curved line (µm)		40.8 ± 9.8	48.2 ± 2.4	0.250	26.6 ± 5.4	27.5 ± 3.4	0.565
Distance straight line (µm)		24.5 ± 5.3	27.6 ± 1.6	0.412	12.5 ± 1.7	13.5 ± 2.3	0.313
Distance average path (µm)		27.6 ± 6.2	31.2 ± 1.4	0.409	14.7 ± 2.2	15.6 ± 2.4	0.355
Velocity curved line (µm/s)		102.7 ± 5.3	110.0 ± 4.8	0.051	64.3 ± 12.7	67.02 ± 5.9	0.569
Velocity straight line (µm/s)		64.5 ± 4.4	65.2 ± 2.5	0.802	34.9 ± 4.2	37.2 ± 3.7	0.434
Velocity average path (µm/s)		71.1 ± 3.3	72.6 ± 2.0	0.560	39.3 ± 5.4	41.7 ± 3.9	0.459
Beat cross frequency (Hz)		19.0 ± 1.9	21.3 ± 0.8	0.172	9.3 ± 2.8	10.8 ± 1.5	0.249
Amplitude of lateral head displacement (µm)		*0.81* ± *0.09*	*0.91* ± *0.07*	*0.028*	0.61 ± 0.11	0.63 ± 0.06	0.612
Progressive motility (%)	168	*68.9* ± *1.3*	*49.9* ± *12.4*	*0.044*	32.8 ± 5.6	20.9 ± 9.7	0.061
Distance curved line (µm)		*40.3* ± *6.5*	*26.8* ± *8.3*	*0.018*	*21.6* ± *3.0*	*12.6* ± *4.6*	*0.019*
Distance straight line (µm)		*22.5* ± *1.4*	*13.7* ± *3.3*	*0.011*	*10.7* ± *1.2*	*4.7* ± *1.3*	*0.007*
Distance average path (µm)		*25.6* ± *2.1*	*15.7* ± *4.0*	*0.009*	*12.3* ± *1.3*	*6.0* ± *1.8*	*0.008*
Velocity curved line (µm/s)		*95.0* ± *15.9*	*65.0* ± *18.9*	*0.014*	*57.2* ± *9.3*	*40.3* ± *13.9*	*0.038*
Velocity straight line (µm/s)		*56.7* ± *4.1*	*36.9* ± *7.4*	*0.005*	*34.0* ± *5.4*	*23.1* ± *4.4*	*0.014*
Velocity average path (µm/s)		*63.0* ± *6.0*	*41.1* ± *9.0*	*0.006*	*37.5* ± *5.8*	*25.9* ± *5.7*	*0.016*
Beat cross frequency (Hz)		*16.7* ± *2.0*	*10.6* ± *3.0*	*0.014*	7.3 ± 1.0	4.0 ± 1.9	0.053
Amplitude of lateral head displacement (µm)		*0.81* ± *0.12*	*0.64* ± *0.14*	*0.017*	0.55 ± 0.04	0.42 ± 0.12	0.079

Significant differences are marked with italics.

## Discussion

Recently, there have been repeated studies in which fertility failures in pig farms were attributed to the use of plastic bags^3,4^. Boar semen bags or blisters are normally made from multilayer plastic films which consist of two or more plastic layers which are glued together with an adhesive to achieve a stable bag with good mechanical resistance and sealing properties (Fig. 1). The most widely used adhesives contain various phthalate esters as plasticizers. These additives have long been suspected to have a negative effect on spermatozoa and living organisms^9,10^. Besides the additives, other non-intentionally added substances (NIAS), such as impurities from starting materials or by-products from the manufacturing process, are also raising safety concerns^11^. The EU regulations classify phthalates as potentially teratogenic substances which can impair fertility (EC Regulation 1935/ 2004, EU Commission regulation 10/2011) but the adhesives used to manufacture multilayer plastic materials are not well regulated^12,13^.

Plastic materials used for semen packaging are mostly made from polyolefins that offer great flexibility, mechanical strength, low weight, stability, high density and low cost compared to other materials^14^. This group includes polyethylene (HDPE or LDPE), polypropylene (PP), polyvinylchloride (PVC), polyethylene terephthalate (PET) and other copolymers such as ethylene vinyl acetate (EVA)^15^. Currently, there are two types of manufacturing processes used to produce boar semen packaging units. The most commonly used type of semen container is the boar semen tube^16–18^ made of pure polyethylene. Polyethylene semen tubes are processed using a blow-molding machine that melts plastic pellets and forms the semen tube or bottle. No additives are needed for this straightforward production system.

In contrast, boar semen bags are manufactured with the abovementioned multilayer plastic films involving the use of risky raw materials like adhesives that may or may not have adequate quality control measures in place. Nerin et al. (2014) elucidated in their study the origin and reasons behind a dramatic reproductive failure in more than 40 Spanish sow herds in the spring of 2010^4^. The chemical analyses of the used plastic bags revealed five different toxic compounds: BADGE, BADGE-H_2_O, BADGE-2H_2_O, cyclic lactone and cyclic phthalates. BADGE is a derivate of Bisphenol A and long suspected to interfere with human male fertility^19^. The origin of these toxic compounds was found to be the adhesive used to manufacture the multilayer plastic bags. The study also revealed that the toxic compounds of the plastic bags leach through intact plastic films into the extended semen and react there with the water molecules to form new compounds; namely BADGE-H_2_O and BADGE-2H_2_O^20,21^.

The amount of BADGE detected by Nerin et al. in 2014^4^ in the migration study was in a similar range compared to our study (0.40 ± 0.14 mg/L and 0.37 ± 0.05 mg/L, respectively). Other compounds, like cyclic lactones or phthalates, could not be found in the present migration study. The total concentration of BADGE compounds and their derivatives detected by the chemical analyses conformed to the relevant European Regulation 10/2011/EU for food safety, which allows a maximum value of 9 mg/kg food. Contrary to our study, no spermatological alterations or cell toxicity was observed in the former study and the research group concluded that other characteristics should be investigated to elucidate the mechanism of action of these toxic compounds.

The current study shows that sperm defects may be induced following exposure to BADGE and could produce fertility-relevant malfunctions. The findings lead us to the hypothesis that the BADGE damage of sperm may be in addition to the stage of blastocyst implantation or later stages of fecundation as previously described^4^. This is plausible since it has been shown previously in reproductive toxicological studies on rabbits, that there was no effect on the implantation rate, litter size or intrauterine development following exposure to BADGE^22^. We hypothesize that BADGE and/or BADGE derivatives affect sperm functionality. Especially an extension of the semen storage period and/or incubation time at body temperature (e.g. thermo-resistance test) showed the potential of an early diagnosis. Thermo-resistance test simulates the time of spermatozoa in the female genital tract by exposure to 38 °C for a long time (e.g. 300 min) and concomitantly sperm motility after this heat stress is measured. After a prolonged storage and incubation time, only spermatozoa with a functional metabolism are motile (shown as high persistency).

Leaching processes depend on a variety of factors including contact time, temperature and initial concentration of the compounds in the plastic material^4^. For this reason, the transport of toxic compounds will be higher in the semen doses stored for longer periods in plastic bags or in bags with a higher concentration of the leachable toxic compound. After 168 h of semen storage, total and progressive motility as well as nearly all kinematics were negatively influenced by the storage in the tested bags compared to the controls. There were noted differences in sperm velocity, beat cross frequency and amplitude of lateral head displacement showed a weakened motility pattern and is plausible this would make passage through the utero-tubal junction and the attainment of hyperactivity for fertilization rather unlikely.

Additionally, there was a negative effect on sperm quality as observed at the mitochondrial (energy machinery) and plasma membrane (and acrosome) level of spermatozoa following exposure to BADGE. Furthermore, these sperm quality characteristics are directly related to the fertilisation capacity of an ejaculate^23,24^ and should be incorporated into future QA programs. Hence, it may be possible to prevent fertility failures using a spermatological assay panel to detect sub-optimal semen doses and prevent their use for AI. In this context, further research is needed to investigate the effect of BADGE on sperm capacitation and its influence on fertility when it is disrupted. Sophisticated spermatological assays seem to be more meaningful than complex and expensive chemical studies and allow one to establish a cost-effective and timely QA program before using a given batch of plastic consumables in boar semen production. Classical spermatological methods such as sperm motility after short storage periods, morphology or damage to sperm chromatin structure may not play a key role in BADGE-caused fertility failures. The results obtained in the present study could help to understand the interaction of potentially toxic compounds with spermatozoa, as seen by other authors^25,26^. For example, in human sperm, BADGE may lead to an activation of specific Ca^2+^ channels (CatSper)^27^ that could influence important sperm functions.

In conclusion, direct exposure of boar spermatozoa to toxic compounds is a feasible and cost-effective method for reprotoxic studies on male fertility. Further investigations are needed to determine the optimal spermatological assay panel to be implemented during testing of plastic materials as an integral part of future QA programs in breeding companies.

## Materials and methods

### Chemicals and reagents

Unless specifically stated, the chemicals used in this study were purchased from Roth (Karlsruhe, Germany) and Merck (Darmstadt, Germany) and were of analytical grade. Acridine orange was obtained from Polysciences Europe GmbH (Eppelheim, Germany) and Merocyanin 540 (M540) and YoPro-1 from Molecular Probes (Leiden, the Netherlands). The fluorescent dyes fluorescein-isothiocyanate conjugated peanut agglutinin (FITC-PNA) and *Pisum sativum* agglutinin (FITC-PSA) were purchased from Axxora (Lörrach, Germany). Propidium iodide (PI) and Rhodamine 123 (R123) were obtained from Sigma-Aldrich (Steinheim, Germany).

### Plastic bag samples and fertility data

Fertility data was collected from four farms under the same management in Croatia. The reported fertility data represents similar timeframes for all farms 8 weeks prior (Before), 8 weeks after (After) and 8 weeks during (During) the usage of the questionable semen bags (Table 1). Semen bags (MEDI NOVA, Italy) for all four farms were delivered from the same AI center in Croatia. For the characterization of the plastic bags regarding migration studies and spermatological analyses, eight randomly selected bags were analyzed from the affected batch.

### Migration study of the plastic bags

Migration studies were performed on MEDI NOVA bags (Code: 1908003, MEDI NOVA, Italy) and control bags. The tested bags were new and unused. In each case, eight bags were filled with 30 ml of simulants (10% ethanol and water^2,3^) and placed in a horizontal position so that the liquid was in contact with the entire surface of the bags. Three replicates were prepared. Bags were kept in an oven (HERAEUS B6120, Hanau, Germany) at 37 °C for 72 h. The extracts and blank samples were then simultaneously analyzed by gas chromatography-mass spectrometry (GC–MS/MS) at the Institute for Food and Environmental Research, Potsdam, Germany. The GC–MS was performed on a 6.890 series gas chromatography coupled to a 5.973 mass spectrometer from AGILENT (Palo Alto, CA, USA) using AGILENT CHEMSTATION software. The identification of compounds was verified by known reference standards. Confirmation was done with the pure standards analyzed under the same conditions.

### Boar semen collection and processing

All procedures involving animals were carried out in accordance with guidelines and regulations according to the European Commission Directive for Pig Welfare and were approved by the animal welfare committee of the state of Brandenburg (TVO-2019-V-21). Ejaculates were purchased from a commercial AI center located in the north part of Germany (BHZP, Bösewig, Germany). All boars in this study were routinely used for semen collection and AI dose production. The average age (mean ± SD) of the 16 fertile Pietrain boars included in this study was 16.5 ± 4.3 months. They received commercial feed (pellets) for AI boars and were housed in individual pens equipped with nipple drinkers according to the European Commission Directive for Pig Welfare. The collection frequency of ejaculates did not exceed three collections within two weeks with at least three days of rest in between. Semen production protocols were used according to the general guidelines for semen processing used in AI studs participating in a quality control audit of the Institute for the Reproduction of Farm Animals Schönow^28^. Only ejaculates that passed minimum requirements for commercial use in AI were included. Criteria for the selection of ejaculates comprise a minimum of 75% morphologically normal spermatozoa, at least 70% total sperm motility and a total number of ≥ 30 × 10^9^ spermatozoa per ejaculate.

All ejaculates were collected randomly by the gloved-hand method and processed in one day. The day of collection is specified as Day 0 (d0). The pre-sperm phase of each ejaculate was discarded, and the gel fraction of the semen was removed by gauze filtration during collection. To avoid individual variability between AI boars in each experiment, semen from four different males was pooled (4 × 4 ejaculates, in total 16 ejaculates). Pooled ejaculates were evaluated after semen dose preparation in a split-sample procedure for sperm motility, thermo-resistance, mitochondrial activity, membrane fluidity/integrity and DNA integrity. Ejaculate volume was determined to produce AI doses of 85 ± 1 ml. Sperm concentration was measured using a NUCLEOCOUNTER SP-100 (Chemometec, Denmark) and adjusted to 24 × 10^6^ spermatozoa/ml. Dilution was performed with an isothermic (32 °C) Beltsville Thawing Solution (BTS) extender (Minitüb, Tiefenbach, Germany).

Semen was filled in QUICKTIP FLEXITUBES (Control, Minitüb, Germany) or in MEDI NOVA bags (Bags). Immediately after filling, AI doses were placed in a temperature-controlled box at 21 °C for 90 min (controlled room temperature). The temperature of the box was then reduced to 17 °C and samples were transported to the reference laboratory (overall cooling rate 4 °C/hour), where the samples were stored for 168 h at 17 °C during which they were subjected to further analyses. Except for days on which analyses occurred, there was no rotation of samples.

### Evaluation of sperm morphology

To assess sperm morphology after 24 and 72 h of semen storage, spermatozoa were fixed by 1% formalin in phosphate-buffered saline at a concentration of 50–100 × 10^6^ spermatozoa/ml. Two hundred spermatozoa per sample were evaluated using phase contrast microscopy (800 × total magnification, Jenaval, Carl Zeiss Jena, Germany). The percentage of morphologically normal spermatozoa and further subcategories was evaluated according to Schulze et al*.*^29^.

### Evaluation of sperm motility and thermo-resistance

Sperm motility and kinematic data were assessed after 24, 48, 96, 120 and 168 h of semen storage using the computer-assisted sperm analysis (CASA) system ANDROVISION (Minitüb, Tiefenbach, Germany) according to methods described previously^30^. Prior to analysis, a 1.5 ml aliquot of semen from every AI dose was incubated for 10 min at 38 °C.

To assess sperm longevity at body temperature, a thermo-resistance test (TRT) was performed after 24 and 168 h of semen storage as described previously^18^. Based on this procedure, 10 ml aliquots from AI semen doses from each trial group were incubated in a water bath (GFL 1002, Gesellschaft für Labortechnik, Burgwedel, Germany) at 38 °C for up to 300 min. Measurements of sperm motility and kinematics were performed after 30 and 300 min of incubation time. Spermatozoa were defined “motile” when showing an amplitude of lateral head displacement (ALH) < 1.0 µm and a velocity curved line (VCL) < 24.0 µm/s.

### Flow cytometric assessment of boar spermatozoa

Analyses of mitochondrial activity, membrane integrity and fluidity were performed using a CYTOFLEX S FLOW cytometer (Beckmann Coulter, Krefeld, Germany) equipped with a 405 nm, 488 nm, 561 nm and 638 nm solid-state laser. For evaluation of chromatin structure an ACCURI C6 FLOW cytometer (BD Accuri, Fa. BD Biosciences, Erembodegem, Belgium) was used equipped with a 488 nm solid-state laser. The sperm population was gated referring to the expected forward- and side-scatter signals. For each sample evaluated, a total of 30,000 events fitting the respective gate were counted. Samples were incubated at 38 °C using a dry block heater (TECHNE DRI-BLOCK DB2.D, Techne AG, Burkhardtsdorf, Germany).

### Evaluation of mitochondrial activity

Sperm viability and mitochondrial activity were assessed after 168 h of semen storage by double-staining with R123/PI and flow cytometry as described previously^24^. Fluorescence signals of R123, gathered via 533/30 nm band-pass filter, and PI, gathered via 670 nm long-pass filter, were plotted on logarithmic scales. The viable sperm subpopulation with active mitochondria (R123 pos. and PI neg.) was determined as percent from overall sperm population.

### Evaluation of plasma membrane and acrosome integrity

A triple-stain flow cytometric method using PI, FITC-PNA, and -PSA fluorescent dyes was used after 168 h of semen storage to discriminate between viable (intact plasma membrane) and dead spermatozoa and to characterize membrane integrity in the acrosomal region as described previously^30^. Fluorescence signals of FITC-PNA and FITC-PSA, were collected via 533/30 nm band-pass filter, while PI was collected via a 670 nm long-pass filter. The fluorescent signals for all assays were plotted on logarithmic scales. Sperm subpopulations with intact plasma membranes and intact acrosomal membranes (i.e. PI neg., PNA neg. and PSA neg.) were determined as percent from overall sperm population.

### Evaluation of membrane fluidity

Sperm plasma membrane lipid organization and sperm viability were assessed simultaneously after 168 h of semen storage by staining with M540 and YoPro-1, respectively, using a 488-nm laser. Samples were incubated with YoPro-1 (75 nM) and M540 (6 µM) at 38.5 °C for 15 min. The M540 probe exhibits high affinity for membranes with increased lipid disorder^31^, emitting fluorescence that is detected with a 572 ± 28 nm band pass filter. Sperm viability was evaluated with YoPro-1, a non-permeable viability stain that penetrates spermatozoa with damaged membranes, showing green fluorescence that is detected with a 530 ± 30 nm band-pass filter. Both were plotted on logarithmic scales. Results are expressed as the percentage of viable cells (YoPro-1 negative) with high M540 fluorescence (M540 positive cells), viable cells with low M540 fluorescence (M540 negative cells) and dead spermatozoa (YoPro-1 positive).

### Evaluation of sperm chromatin structure

For evaluation of sperm DNA integrity, a sperm chromatin structure assay was performed after 72 h of semen storage in diluted semen samples according to Evenson and Jost^32^. Briefly, 200 µl TNE-buffered aliquots of semen samples were supplemented with 400 µl of acid solution, mixed on a vortex for 30 s and incubated with 1.2 ml of staining solution (300 µl 0.1% acridine orange solution in 50 ml *aqua bidest.*) on iced water for 3 min in the dark. Fluorescence signals of acridine orange were gathered via 670 nm long-pass filter and via 533/30 nm band-pass filter as forward- and side-scatter signals and plotted on linear scales. The DNA-fragmentation index (DFI) was calculated as the quotient of red fluorescence to total fluorescence and gives the percentage of spermatozoa with fragmented DNA.

### Statistical analysis

Data analysis was performed using SPSS Statistics 23 (IBM, Armonk, USA) and R (R Foundation for Statistical Computing, Vienna, Austria)^33^. All descriptive data are expressed as mean ± standard deviation (SD). While only four pairs of samples were compared, Student’s t-Test was originally created to handle similar small sample sizes^34^. We therefore used the paired t-test for comparisons of spermatological data between bags and controls at each time point. The development of sperm motility from 24 to 168 h of semen storage was evaluated using Kendall’s τ correlation. Statistical analysis of fertility data was performed by two-way analysis of variance (ANOVA, 2 × 2) followed by Fisher's Least Significant Difference (LSD) test. Differences among means were considered significant at *P* ≤ 0.05.
